# High prevalence of mutations in peripherin/RDS in autosomal dominant macular dystrophies in a Spanish population

**Published:** 2007-06-28

**Authors:** María José Gamundi, Imma Hernan, Marta Muntanyola, María José Trujillo, Blanca García-Sandoval, Carmen Ayuso, Montserrat Baiget, Miguel Carballo

**Affiliations:** 1 Servicio de Laboratorio. Biología y Genética Molecular Hospital de Terrassa, Ctra. Torrebonica,Terrassa, Barcelona, Spain; 2 Servicio de Genética Fundacion Jiménez Díaz CIBER-ER ISCIII Madrid, Spain; 3 Servicio de Oftalmología Fundación Jiménez Díaz CIBER-ER ISCIII Madrid, Spain; 4 Servicio de Genética Hospital de la Santa Creu i Sant Pau. CIBER-ER ISCIII Barcelona, Spain

## Abstract

**Purpose:**

Mutations in the peripherin/retinal degeneration slow (RDS) gene are a known cause of various types of central retinal dystrophies. The purpose of this study was to determine the prevalence of mutations in the peripherin/RDS gene in Spanish patients with different types of autosomal dominant macular dystrophy.

**Methods:**

Ophthalmic and electrophysiological examination was performed in patients from 61 unrelated autosomal dominant macular dystrophy (adMD) Spanish families. Screening for mutations in the peripherin/RDS gene by denaturing gradient gel electrophoresis (DGGE) and direct genomic sequencing was performed in index patients and extended to the family when positive.

**Results:**

We report four novel mutations in peripherin/RDS and a relatively high frequency (23%) of mutations in this gene in families with adMD. Thirteen different mutations were found in fifteen adMD families. Three novel missense, four nonsense and a cis-acting splicing mutation IVS2+2T>C, were found in a Spanish population while five more missense mutations were also reported in other populations. The Arg142Trp and Arg172Trp mutations, present in several populations, were both detected in two independent Spanish families. All the missense mutations produce an amino acid substitution in the second intradiscal loop of the peripherin, while the nonsense mutations presumably generate a truncated protein.

**Conclusions:**

A high frequency (23%) of mutations in the peripherin/RDS gene was found in a cohort of 61 unrelated patients with various types of autosomal dominant central retinal dystrophies as compared with a low prevalence (1.3%) of mutations in this gene causing retinitis pigmentosa in a Spanish population. Different macular dystrophy phenotypes according to the mutations in peripherin/RDS are shown. However, a limited phenotype variation was observed for these mutations within the family.

## Introduction

The peripherin/RDS gene encodes a glycoprotein which is confined to the outer segment disk of both rod and cone photoreceptor cells [1,2]. The normal product of this gene, peripherin, interacts in vivo with rod outer segment membrane protein 1 (ROM1) in rods and plays an important structural role in photoreceptor outer segments [3]. Over 70 mutations in the peripherin/RDS gene have been reported in autosomal dominant retinitis pigmentosa (ADRP) and autosomal dominant macular dystrophy (adMD) [4-9], including a digenic trait with the ROM1 gene [10]. Phenotypic variation has been observed with different mutations in this gene, with an extreme case of variable phenotypes within a family having a single mutation showing retinitis pigmentosa (RP) and macular dystrophy (MD) [11,12]. In our previous screening for mutations in the peripherin/RDS gene and in other RP and MD related genes in index cases of autosomal dominant retinopathies and simplex cases of RP, we found a lower contribution (1.3%) of mutations in peripherin/RDS to ADRP [13] in a Spanish population as compared to other screened populations. However, we found a relatively high association (23%) of mutations in this gene in families with autosomal dominant macular dystrophies. As reported in other populations, variability in macular dystrophy phenotype is also observed in this Spanish population. We report the different macular dystrophy phenotypes associated with mutations in the peripherin/RDS gene found in a Spanish population and compare these phenotypes with the associated mutations reported in other populations.

## Methods

A cohort of 61 unrelated patients with various types of autosomal dominant macular dystrophies, aged between 26 and 75 years, participated in this study. Patients were recruited from Fundación Jiménez Díaz (Madrid, Spain), Hospital de la Santa Creu i Sant Pau (Barcelona, Spain) and Hospital de Terrassa (Barcelona). A control group of 137 non-affected individuals was tested. Informed consent was obtained from all subjects who participated in the study and the research adhered to the tenets of the Declaration of Helsinki.

### Ophthalmologic and electrophysiological studies

All individuals quoted above received a complete ophthalmic examination, which consisted of best corrected visual acuity with Snellen optotypes, color vision with Farnsworth 32 hue test, computerized perimetry (recorded on the Octopus 500) and biomicroscopy and fundus examination after pupillary dilation. Electroretinograms (ERG) and electrooculograms (EOG) were performed according to the standard testing protocols proposed by ISCEV [14].

### Polymerase chain reaction

Genomic DNA was prepared from peripheral blood lymphocytes using QIAmp DNA Blood Mini Kit (Qiagen, Valencia, CA;). Flanking intronic and coding regions of exons 1 and 2 of the peripherin/RDS gene were amplified using the primers shown in Table 1. One PCR primer in each pair included a 40-base GC-rich segment ("GC-clamp") attached to its 5' end to facilitate detection of mutations by denaturing gradient gel electrophoresis (DGGE). PCR reactions were performed in a 50 ml volume of buffer (20 mM Tris-HCl, pH 8.55, 16 mM (NH)_2_SO_4_, 1.5 mM MgCl_2_ 150 mg/ml BSA, and 10% DMSO) containing 50-200 ng of human genomic DNA, 25 pmol of each primer, 10 nmol of each deoxyribonucleoside triphosphate, and 1.5 units of Taq polymerase (Ecotaq, Barcelona, Spain). Incubation was performed for 40 cycles consisting of 30 s at 94 °C, 30 s at 60 °C (for exons 1A and 1B), 63 °C (for exon 2) or 54 °C (for exon 3), and 30 s at 72 °C. This was followed by 5 min at 94 °C and 5 min at 72 °C. Electrophoresis of 8 ml of final PCR reaction volume was performed on 1.5% agarose gel to test the amplification reaction.

**Table 1:** Primers and conditions used for mutation detection.

**Primer**	Sequence* (5'-3')	**Annealing temperature (°C)**	**Amplicon size (bp)**	**DGGE gradient**
Exon 1A	F: (GC)-GGAAGCAACCCGGACTACAC	60	379	40-70%
	R: TAGCCAGGTACGGCTTCAGC			
Exon 1B	F: (GC)-ATTGCATGGAAGCCCTG	60	379	40-70%
	R: TCTGACCCCAGGACTGGAAG			
Exon 2	F: (GC)-AAGCCCATCTCCAGCTGT	63	353	45-75%
	R: CTTACCCTCTACCCCCAGCTG			
Exon 3	F: AGATTGCCTCTAAATCTCCT	54	294	-
	R: GGAGTGCACTATTTCTCAGT			

Within the table, (GC) represents: 5'- CCG CCG CGC CCG CGC CCG GCC CGC CGC CCC CGC CCG-3'. DGGE means denaturing gradient gel electrophoresis. In DGGE gradient column, 100% denaturant equals to 7M urea and 40% (v/v) formamide.

### Mutation detection

PCR-amplified fragments containing the flanking intronic and coding sequences of exons 1 and 2 of the peripherin/RDS gene were analyzed. Exon 1 was divided into two PCR fragments, 1A and 1B. Screening for mutations was carried out by DGGE [15,16]. Electrophoretic conditions (running temperature and denaturing gradient of formamide/urea concentration range for each different PCR product) are shown in Table 1. The PCR-amplified DNA fragment containing exon 3 of peripherin/RDS was sequenced directly because it is difficult to analyze by DGGE. When DGGE variants were observed, the corresponding PCR fragment was sequenced. For DNA sequencing, PCR products were purified using Qiaquick Gel Extraction Purification Kit (Qiagen). DNA sequencing was carried out with the same primers used for amplification with the OpenGene automated DNA sequencing system from Visible Genetics and Thermo Sequenase Cy5.5 Dye Terminator Cycle Sequencing Kit (Amersham Pharmacia Biotech, Barcelona, Spain). Prediction of the pathogenesis of the missense mutations was performed using PolyPhen (Polymorphism phenotyping) program from the Harvard University.

## Results

Out of a total of 61 families studied, we found 13 different mutations in peripherin/RDS in 15 families with adMD (Table 2). These mutations were absent in 137 controls used in peripherin/RDS mutation screening. Co-segregation of the mutation was performed in each family and a complete penetrance was observed in all of them.

**Table 2:** Mutations in the peripherin/RDS gene found in nish patients with autosomal dominant macular dystrophy.

**Exon**	**cDNA change**	**Protein change**	**PSIC score difference**	**Phenotype**	**Reference**
1B	658 TAC>CAC	Tyr141His	2.148	AVDM	[27]
1B	661 CGG>TGG	Arg142 Trp	2.118	CACD	[27,28]
1B	678 del T	Gly148AlafsX152	-	CACD	[29]
1B	751 CGG>TGG	Arg172Trp	2.606	CACD	[4,21-25]
2	821 CGA>CTA	Arg195Leu	2.745	PMD	[30]
2	846_862 del	Tyr204Pro fsX211	-	PMD	[29]
2	860 GGC>GAC	Gly208Asp	1.843	PMD	[29,31]
2	875 TGC>TTT	Cys213Phe	3.665	AVMD	Present study
2	878 TGC>TAC	Cys214Tyr	3.665	PMD	[27]
2	895 CGG>TGG	Arg220Trp	2.97	BPD	[21]
2	948_959 del	Asp237_Thr240del	-	BPD	Present study
2	975-978 dupGGTG	Arg248Gly fsX301	-	PMD	Present study
Intron 2	IVS2+2 T>C	----	-	PMD	Present study

The following abbreviations were used: adult vitelliform macular dystrophy (AVMD); butterfly-shaped pattern dystrophy (BPD), central areolar choroidal dystrophy (CACD), and pattern macular dystrophy (PMD). Position-specific independent counts (PSIC) score calculates the difference between first and second amino acid variant.

**Figure 1:**
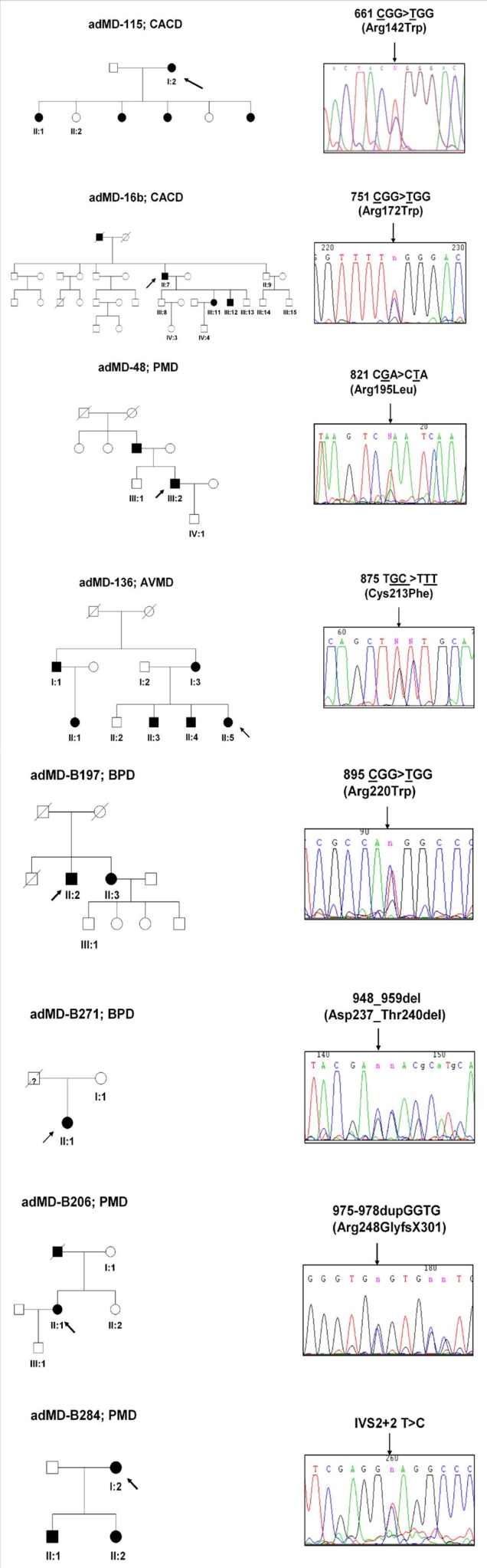
Pedigrees of novel reported Spanish families with mutation in the peripherin/RDS gene. Solid, open, and scored symbols indicate affected, unaffected and deceased individuals, respectively; arrows indicate probands. Bottom numbered symbols in pedigree correspond to individuals whose DNA has been analyzed by DGGE and sequencing- In chromatogram of direct genomic sequencing of mutation, arrows indicate position of mutation. AVMD indicates adult viteliform macular dystrophy; BPD indicates butterfly-shaped pattern dystrophy; CACD indicates central areolar choroidal dystrophy; PMD indicates pattern macular dystrophy.

Eight previously unreported families, are shown in Figure 1. Two of these mutations, Arg142Trp and Arg172Trp, were detected in two unrelated Spanish families. Seven mutations found in the peripherin/RDS gene are missense mutations, and three, Tyr141His, Arg142Trp, and Cys213Phe, have only been reported in a Spanish population, while the Arg195Leu, Cys214Tyr, and Arg220Trp mutations have also been found in other populations. Of the nonsense mutations found, two are deletions (678delT and 846_862del), and one is a 4 bp GGTG duplication. Additionally, IVS2+2T>C, a mutation located in the intron 2 flanking splicing region, was found. To predict the possible impact of this mutation in splicing, we used Splice site prediction by neural network. This program predicted a donor site in the exon/intron boundary (GT) of intron 2, with a score of 0.98. We used a cutoff 0.40 for donor or acceptor sites. When mutation IVS2+2T>C occurs, no donor site is predicted in exon/intron boundary of intron 2, suggesting that this mutation probably abolishes the canonical splice site.

The nonsense and splicing mutations identified are only reported in a Spanish population. These nonsense mutations presumably generate truncated proteins.

**Figure 2:**
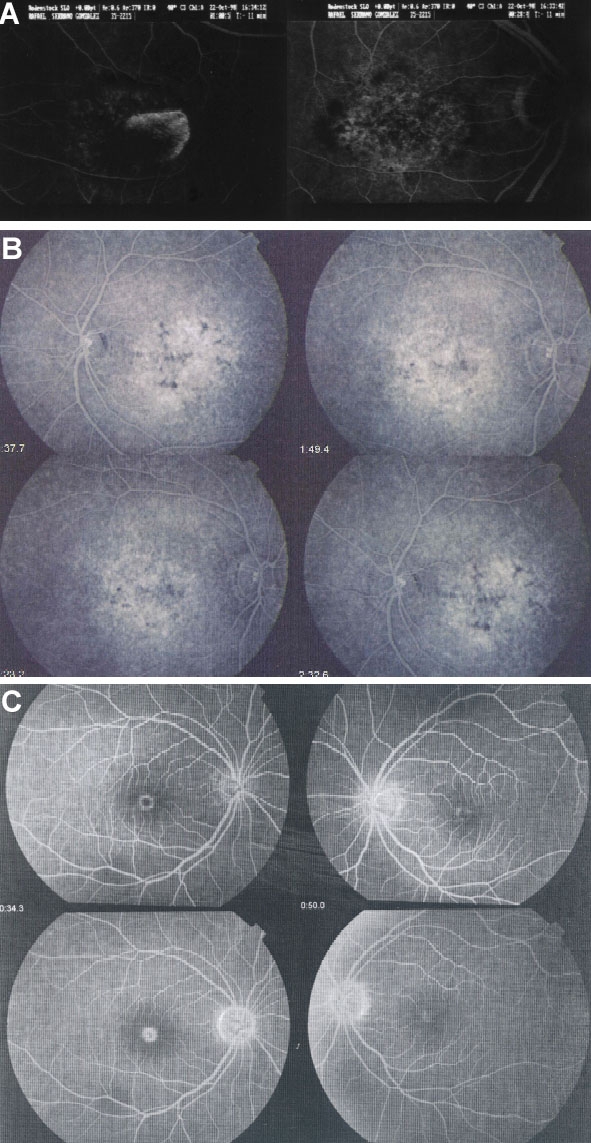
Funduscopic pictures of the different patterns observed in Spanish families with autosomal-dominant macular dystrophy families of a Spanish population. **A**: Central areolar choroidal dystrophy (CACD) caused by mutation Arg142Trp in index patient (male, 39 years old) of family adMD-25. **B**: Pattern macular dystrophy (PMD) present in index patient (male, 33 years old) of family adMD-48, carrier of mutation Arg195Leu. **C**: Adult vitelliform macular dystrophy caused by mutation Cys213Phe in patient II.4 (male, 34 years) of family adMD-136.

Ophthalmic examination and clinical studies of patients were performed according to previously established protocols. Different phenotypes, including central areolar choroidal dystrophy (CACD), adult vitelliform macular dystrophy (AVMD), and pattern macular dystrophy (PMD; Figure 2) were found for these peripherin/RDS mutations. Table 3 summarizes the clinical features of the families.

**Table 3:** Summary of clinical aspects in mutated families.

**Mutation (family)**	**Onsent of vision loss (years)**	**Age at visual acuity <1/10 (years)**	**Central VF loss**	**Photo/Metam**	**Macula**	**ERG**
Tyr141His (DM-24)	40	---	+	+/-	Yellowish lesions at macullae. Atrophy and destructuration of RPE. AVMD	decrease amplitude "b" wave in rods, mixed and cones
Arg142Trp (DM-25)	35	50	+		Subretinian yellowish spots at macullae. RPE destructuration and atrophy. CACD	
Arg142Trp (DM-115)	37	55	+		CACD	decrease "b" wave in cones
Gly148Ala fsX152 (DM-3)	40-45	50	+	-/-	Subretinian yellowish round lesions. RPE destructuration and atrophy. CACD	decrease "b" wave in rods, mixed and cones
Arg172Trp (DM-15b)	40	60	+	+/+	Irregular pigmentation. Demarcated atrophy of central retina. CACD.	Normal
Arg172Trp (DM-16b)	35-55	60-75	+	+/+	Demarcated atrophy of central retina. CACD.	Normal
Arg195Leu (DM-48)	14	26	++		Yellow deposits. Destructuration of RPE. PMD.	Not Performed
Tyr204ProfsX211 (DM-2)	50-70	55-75	+/-	+/+	Yellow deposits. Destructuration of RPE. PMD.	Not Performed
Gly208Asp (B-263)	36		+	-/+	RPE atrophy Flecks in macula. PMD.	Photopic: Normal; Scotopic: Normal
Cys213Phe (DM-136)	34	34	+	+	AVMD	Normal RE decrease "b" wave in cones LE
Cys214Tyr (DM-13)	35		-	-/+	Starred aspect and destructuration of RPE. PMD.	Not Performed
Arg220Trp (B-197)	45		+	Total central visual loss	BPD	Photopic: Normal; Scotopic: Normal
Asp237_Thr240del (B-271)	43		-	+/+	BPD	Photopic: Normal; Scotopic: Diminished
Arg248GlyfsX301 (B-206)	36			-/+	Macula RPE alteration. PMD.	Photopic: Normal; Scotopic: Normal
IVS2+2T>C	48 years		+	-/-	Central RPE atrophy. PMD.	

AVMD indicates adult viteliform macular dystrophy; BPD indicates butterfly-shaped pattern dystrophy; CACD indicates central areolar choroidal dystrophy, ERG indicates electroretinogram, LE indicates left eye, Metam indicates metamorphosia, N.A indicates not available, Photo indicates photophobia, RE indicates right eye, V.F indicates visual field.

## Discussion

Peripherin/RDS is an integral membrane protein essential to outer segment disk morphogenesis of rod and cone photoreceptor outer segments in the retina [1-3,17-19]. Mutations in the peripherin/RDS gene cause a large variety of autosomal dominant retinal degenerations, ranging from RP to MD [4].

In a Spanish population of nearly 200 index cases with ADRP, we detected a lower frequency (1.3%) of mutations in peripherin/RDS than in other reported populations [13]. However, we observed a relatively high frequency (23%) of mutations in peripherin/RDS among autosomal dominant Spanish families with central retinal dystrophies (adMD), slightly higher than that seen in other studies of adMD (18% of AVMD) [20], and 7.3% of adMD in a British population [21].

Specific mutations in the peripherin/RDS gene may lead to a wide inter- and intra-familial variability of phenotypes. Although phenotype-genotype correlations have been suggested depending on the location and type of mutations in peripherin/RDS, a general rule cannot yet be established. However, a common phenotype of adMD has been described for the most reported mutation, Arg172Trp, found in British, Swiss, Swedish, Japanese, and Spanish populations [21-25]. But for the Cys214Tyr mutation, an MD phenotype has been found in one of the Spanish families, while a mutation in the same codon but causing a different amino acid residue substitution, Cys214Ser, has been previously reported to be associated with an RP phenotype [26].

Most pathogenic mutations associated with human retinal dystrophies alter a conserved extracellular/intradiscal domain, EC2, in the protein. Studies carried out by Goldberg et al. [17] showed that changes within the EC2 domain may cause either gross protein misfolding as well as a reduction in protein sedimentation coefficient, while mutations outside the EC2 domain do not seem to affect the protein folding and tetrameric subunit assembly formed by peripherin/RDS and ROM1 protein. All mutations described here lie in EC2, or they produce a predictable truncated protein within this domain. This finding, together with the experimental results observed with peripherin/RDS mutants, suggest a pathogenic mechanism associated with misfolding or reduction of tetrameric subunit assembly in cones that leads to macular degeneration in the retina. Research carried out with animal models by Kedzierski et al [18] studying *rds* and *rom1* transgenic/knockout mice found that photoreceptor degeneration in peripherin/RDS-mediated RP appears to be caused by a simple deficiency of *rds* and *rom1*. Thus, below a critical threshold for the combined abundance of rds and rom1, the extent of outer segment (OS) disorganization results in clinically significant photoreceptor degeneration. In this case, a general haploinsufficiency mechanism could be hypothesized, that isspecially associated with mutations producing null alleles. However, in our studies, the severity and phenotype of the two frameshift changes reported (857del17bp, causing PMD, and 689delT, causing CACD) are not well correlated with the type of mutation and they produced different types of retinal affectation. Although a haploinsufficiency mechanism mediated by peripherin/RDS mutations could not be discarded, more plausible is a pathogenic mechanism that involves a negative dominant effect that could be modulated by other genetic elements that can contribute to the observed heterogeneity of retinal disease phenotypes.

The present study confirms that phenotype-genotype correlation is only feasible for a restricted number of peripherin/RDS mutations, including the most frequently observed Arg172Trp. More descriptions are needed to provide more clues to understanding the underlying mechanisms of photoreceptor degeneration as a consequence of peripherin/RDS mutations

In conclusion, we report four novel mutations and observed a relatively high incidence of mutations in the peripherin/RDS gene in a population of Spanish families with autosomal dominant macular dystrophies with variable phenotypes. However, a relatively homogeneous intra-familial expression of the disease was noted.
